# The Female Pattern Hair Loss: Review of Etiopathogenesis and Diagnosis

**DOI:** 10.1155/2014/767628

**Published:** 2014-04-09

**Authors:** Anja Vujovic, Véronique Del Marmol

**Affiliations:** Department of Dermatology, Hôpital Erasme, Université Libre de Bruxelles, Route de Lennik 808, Anderlecht, 1070 Brussels, Belgium

## Abstract

Female pattern hair loss (FPHL) is the most common hair loss disorder in women. Initial signs may develop during teenage years leading to a progressive hair loss with a characteristic pattern distribution. The condition is characterized by progressive replacement of terminal hair follicles over the frontal and vertex regions by miniaturized follicles, that leads progressively to a visible reduction in hair density. Women diagnosed with FPHL may undergo significant impairment of quality of life. FPHL diagnosis is mostly clinical. Depending on patient history and clinical evaluation, further diagnostic testing may be useful. The purpose of the paper is to review the current knowledge about epidemiology, pathogenesis, clinical manifestations, and diagnosis of FPHL.

## 1. Definition of FPHL

The FPHL is a* nonscarring* progressive thinning of hair. It results from a progressive decrease in the ratio of terminal hairs to shorter, thinner vellus hairs, a process known as follicular miniaturization [1]. This miniaturization follows usually a pattern distribution. In women, FPHL typically presents as a diffuse reduction in hair density over the frontal and vertex areas, but parietal and occipital regions may be involved [2].

## 2. Terminology

In the past, the term “androgenetic alopecia” (AGA) was the primary term used to refer to this condition in both men and women. The term “andro” from ancient Greek refers to male subjects and “genetic” referred to the contribution of heredity. Over the years, “female pattern hair loss” became the preferred term for this form of hair loss. This terminology helps to distinguish the different features of the condition in women versus men and shows the lack of clear hormonal contribution in many cases. Further, some authors use the terms “androgen-dependent FPHL” and “androgen-independent FPHL” to separate women who have FPHL due to androgen excess from those with normal androgen levels [3].

## 3. Epidemiology

FPHL is very common and increases with age in the Caucasian women populations. In 2001, Norwood established the prevalence of FPHL at 19 percent in a series of approximately 1000 Caucasian women [4]. Although FPHL can occur at any time of life, the condition occurs the most following menopause. This age-related rise was clearly established in Norwood's series; FPHL was only detected in 4 of 121 women between the ages of 20 and 29 (3 percent), but in 41 of 140 women between the ages of 70 and 89 (29 percent) [4]. In a British study of 377 women, 38 percent of women over the age of 70 years had FPHL [5]. The prevalence of the condition seems to be lower in the Asian population [6, 7]. A Korean study shows that the prevalence of FPHL in Korean women at all ages was only 5.6%. Like in Caucasian women, this prevalence increases steadily with advancing age [7]. There are no published data over the prevalence of FPHL in African women.

## 4. Etiology and Pathogenesis

The visible thinning of hair of the scalp in FPHL results from a progressive decrease in the ratio of terminal hairs to shorter vellus hairs, a process called follicular miniaturization [8]. The mechanism through which this follicular transformation occurs in FPHL is not completely understood. Although the roles of androgens and genetic susceptibility in male AGA are well accepted, the degree to which these factors contribute to FPHL is less clear.

### 4.1. Androgens

**Figure 1:**
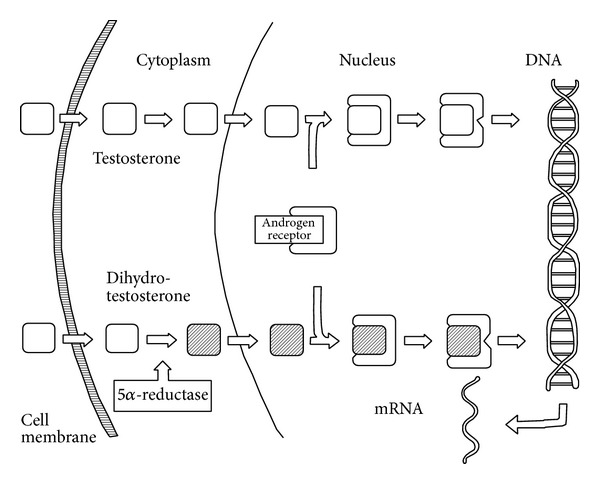
Schematic of the general mechanism of androgens action. Inside the cell, testosterone and DHT bind to the androgen receptor. Once the hormone has bound, the complex will bind to the DNA, altering the expression of specific androgens-dependent genes [10]. Reproduced by Thierry Huart.

The androgens role in common male baldness was first suspected by Hamilton in 1942. He notes that AGA does not occur in men who have never entered puberty, that baldness stops its progression in castrated men, and that testosterone replacements stimulate progressive balding [9]. In recent studies, AGA is described as a consequence of the direct effects of dihydrotestosterone (DHT) on the dermal papilla of susceptible hair follicles [10]. DHT is a more potent androgen, coming from the metabolism of testosterone by the action of the 5*α*-reductase. DHT binds to androgen receptors in hair follicles, more strongly even than testosterone, resulting in upregulation of genes responsible for the gradual transformation of terminal hair follicles to miniaturized hair follicles (Figure 1) [11]. These miniaturized hairs of various lengths and diameters are the hallmark of FPHL and AGA [10, 12, 13]. However, the number of follicles per unit area remains the same [14]. The pattern of hair loss, which typically spares the occipital scalp, reflects regional differences in the sensitivity of scalp follicles to androgens. Some authors have theorized that a similar process contributes to the development of FPHL, a concept supported by the observation that women with hyperandrogenism may develop early-onset FPHL [2]. However, most women with FPHL have no other signs or symptoms of hyperandrogenism and have normal androgen levels, indicating that our understanding of the pathogenesis of the disorder remains incomplete. The age-related increase in FPHL and the highest rates in postmenopausal women may suggest a protective role of the estrogen. Supporting this theory, Sawaya and Price conducted a study in 12 young women and 12 young men (ages from 14 to 33) suffering from AGA or FPHL [15]. Scalp biopsies were taken and androgens, expression of androgen receptor, type I and type II 5*α*-reductase, and cytochrome p-450 aromatase enzyme genes were measured in hair follicles. Both young women and young men had higher levels of type I and type II 5*α*-reductase and androgen receptors in frontal hair follicles compared to occipital hair follicles explaining probably the patterned hair loss. However, the levels in women were approximately half the levels in men [15]. The findings of this study suggest that the milder expression of FPHL may in part be the result of lower levels of 5*α*-reductase and androgen receptors in frontal follicles of women compared to levels in men. Additionally, young women had much higher levels of cytochrome p-450 aromatase, enzyme capable of converting testosterone to estradiol, in frontal and occipital follicles than men. Those notable increased aromatase levels seem to play a protective role in the development of hair loss in women [15]. Furthermore, supporting the androgen-dependent etiopathogenesis, low levels of sex hormone-binding protein (SHBG), glycoprotein that binds to androgens, inhibiting thereby their activities, have been linked to diffuse hair loss [16]. Another part of FPHL and AGA pathogenesis is the gradual shortening of the growth phase of hair follicles. Over the successive hair cycles, the duration of anagen phase shortens from a normal duration of a few years to only weeks to months [2].

### 4.2. Genetics

There are few studies evaluating the genetic basis and inheritance pattern of FPHL [8]. One of them shows an incidence of 54% pattern hair loss in first-degree male relatives of age >30 years and 21% in first-degree female relatives >30 years. Those reports of the occurrence of both FPHL and AGA in individual families suggest that FPHL and AGA share a common genetic background [17, 18]. The two major susceptibility loci for the AGA in men are the androgen receptor (AR)/ectodysplasin A2 receptor (EDA2R) locus on the X-chromosome and a locus on chromosome 20p11, for which no candidate gene has yet been identified [19, 20]. Very recent studies show no involvement of the well-established locus on chromosome 20p11 in FPHL but suggested that the X-chromosomal locus containing the androgen receptor (*AR*) and the ectodysplasin A2 receptor (*EDA2R*) genes may be specifically involved in the pathogenesis of early-onset FPHL [21]. Moreover, an Australian genomewide association study suggested that the aromatase gene (CYP19A1) may contribute to FPHL [22].

## 5. Clinical Features

**Figure 2:**
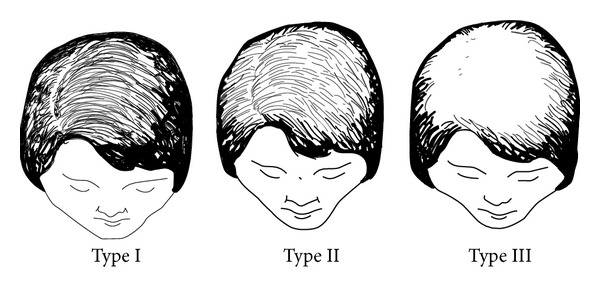
Ludwig pattern of hair loss in women. Three-point scale. Diffuse thinning of the crown region with preservation of the frontal hairline. Drawing by Thierry Huart based on Ludwig et al. [24, 25].

**Figure 3:**
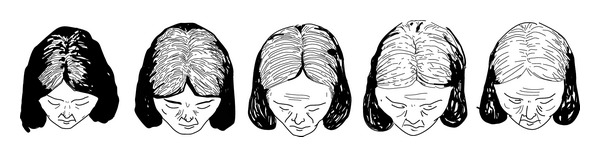
Sinclair scale: 5-point scale for grading of FPHL with diffuse thinning of the crown region with preservation of the frontal hairline. Drawing by Thierry Huart based on the Sinclair Scale, Sinclair et al. [25].

**Figure 4:**
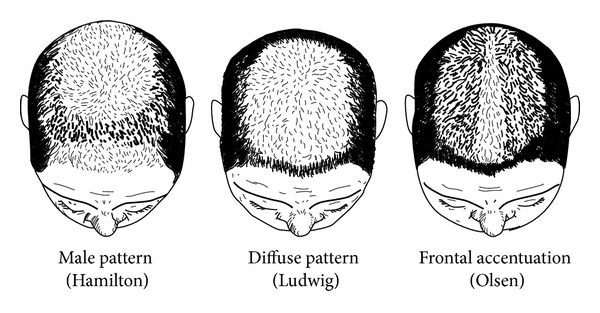
Olsen scale: Christmas tree pattern in female pattern hair loss. Thinning associated with bitemporal recession. Drawing by Thierry Huart based on Olsen scale, Olsen [26].

FPHL may have three different patterns [23]:

1. diffuse thinning of the crown region with preservation of the frontal hairline: two scales are used to describe this pattern: the commonly 3-point Ludwig scale [12, 24] (Figure 2) and the 5-point Sinclair scale [25] (Figure 3);
2. thinning and widening of the central part of the scalp with breach of frontal hairline, described by Olsen scale: Christmas tree pattern (Figure 4) [26];
3. thinning associated with bitemporal recession; Hamilton-Norwood scale [27].

All these common patterns spare the occipital area, a phenomenon explained probably by hormonal influences explained above. This behavior's difference between the frontal/parietal follicles and the occipital follicles is found in other hair disorders like alopecia areata, a condition where occipital follicles affected by the ophiasis pattern are typically more resistant to regrowth [28]. These differences may result from the embryological derivation of the dermis in the two regions. It is known from avian embryology that the dermis of the frontal/parietal scalp is of neural crest origin, whereas the dermis of the occipital scalp is of mesodermal origin [29].

## 6. Associated Disorders

### 6.1. Psychosocial Dysfunction

Many women suffering from FPHL experience negative psychosocial effects related to the condition. In a questionnaire-based study, 70 percent of affected women reported that they were very to extremely upset about their hair loss. They experienced more feeling of negative image body and poorer self-esteem and had a less quality of life than the control group [30]. In another study, 88 percent of females with FPHL felt that the hair loss negatively influenced daily life [31]. Clinicians managing patients with FPHL should remain aware that the patient's perception of the severity of hair loss may be different from the clinical assessment of the severity and the psychosocial impact of the condition. In a recent questionnaire-based study of 104 women suffering from different hair loss disorders like alopecia areata, FPHL, or telogen effluvium, the patient's perception of the severity of hair loss was greater than the severity ratings given by the dermatologist. In addition, the personal rating of hair loss will be more closely correlated with the effects of hair loss on quality of life [32]. This psychosocial dysfunction related to FPHL is also experienced by adolescent girls suffering from the condition. Those teenage girls may experience poor self-esteem and impaired functioning at home, school, or work and in personal relationships [33].

### 6.2. Medical Conditions

Links between early-onset FPHL and insulin resistance and hypertension and increased cardiovascular risk have been described [34, 35]. Those conditions seem to be due to higher aldosterone, C-protein, D-dimers, and insulin levels in women suffering from FPHL than in control subjects. Authors recommend the determination of metabolic syndrome and ultrasound study of the carotid arteries to detect risk of developing cardiovascular disease in female patients with early-onset FPHL [35]. Recently, vertex pattern AGA in male patients of all ages was associated with an increased risk of prostate cancer [36]. There is no evidence in the current knowledge of an association between FPHL and the risk of any kind of cancer.

## 7. Clinical Diagnosis

### 7.1. General History

The physician should record age of onset and duration and progression of hair loss. The patients often describe a chronic hair loss with some increased periods of activity, particularly during autumn and winter. The patient should be asked about thinning and shedding. For thinning, most patients described an accentuation of the frontal, parietal, or vertex region, but a diffuse thinning is possible as well. The family history is more often positive, but a negative family history does not exclude the diagnosis. The patient should be asked about other familial hair disorders like alopecia areata or hirsutism, which may influence the further investigations. It is also important to exclude other causes of hair loss whose untreated presence could affect the efficacy of the FPHL treatment. The presence of other medical disorders and newly diagnosed diseases within one year prior to first signs of hair loss and the medical treatment should be investigated. Other causes of hair loss such as diffuse effluvium due to iron deficiency, infection, thyroid dysfunction, or chronic deficient diet should be excluded. Some drugs such as chemotherapeutic agents, proandrogenic hormones, or antithyroids may cause diffuse hair loss. Moreover, some cosmetic habits (traction) or environmental factors like smoking [37] or UV-exposition [38] may induce increased hair loss in women. The experts all agree to ask about eating behavior, as chronic deficient diet or rapid important weight loss can trigger diffuse effluvium [23].

### 7.2. Gynecological History

A complete gynecological and obstetrical interrogatory that includes menarche, menstrual cycle (regular/irregular), menopause, amenorrhea, use of oral or systemic hormonal contraception, hormone replacement therapy, fertility treatment, problems in getting pregnant, gynecological surgery, pregnancies, births, miscarriages, and signs of hyperandrogenism (excessive body hair growth, acne, ect.) should be done to exclude influencing hormonal deregulations (e.g., hormone sensitive tumor) [39]. Impaired fertility, amenorrhea, irregular menstrual cycle, and signs of hyperandrogenism and hyperseborrhea may be indicative for polycystic ovary syndrome [40].

### 7.3. Physical Examination

A full skin examination that includes body and nails is advised in women complaining of hair loss. Nails abnormalities identification is not typical in FPHL but may differentiate the condition from other cases of hair loss like alopecia areata, iron deficiencies, or lichen planus [23]. A whole body examination should be performed to find other signs of possibly associated hyperandrogenism.

### 7.4. Scalp and Hair Scalp Examination

Scalp examination should focus on identifying the distribution of hair loss and the caliber of hairs in commonly involved areas. Findings consistent with FPHL include the detection of terminal hair loss, variation of hair caliber, and miniaturized hairs. Pull test identifying increased shedding of telogen hairs is typically negative except in active phases of FPHL. The most frequently used scales are the Ludwig (Figure 2) and the Olsen (Figure 4) scales, described previously. FPHL is a nonscarring alopecia, explaining that the scalp skin appears normal, but other clinical features such as inflammation, scarring, or hyperseborrhea can be associated and potentially aggravating the FPHL [41]. The physician should also consider scarring alopecia mimicking FPHL like frontal fibrosing alopecia [42].

### 7.5. Trichoscopy

**Figure 5:**
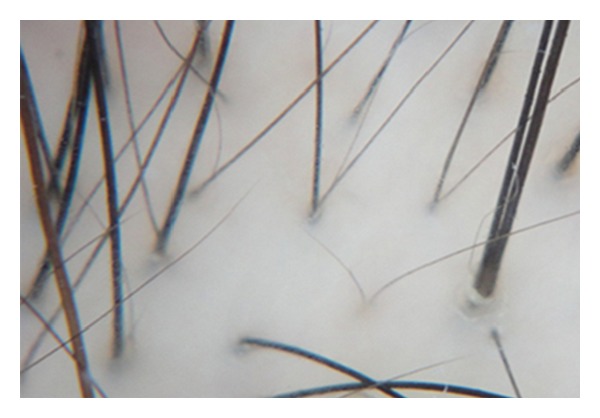
Trichoscopy of the frontal scalp in a female patient complaining of chronic hair loss. Trichoscopy shows FPHL: hair shaft variability greater than 10%, vellus hairs, and perifollicular discoloration.

Trichoscopy or scalp dermatoscopy is a noninvasive diagnostic tool, very useful for the diagnosis and followup of hair and scalp disorders [43]. Trichoscopy of FPHL is characterized by hair diameter variability greater than 20% [44] (Figure 5). Hair shaft variability can also be present in alopecia areata. However, in this pathology dermatoscopy shows uniform miniaturization instead of hair shafts with different degree of thinning. This hair diameter variability is very useful to detect early FPHL in children or teenage girls [45]. In 2009, Rakowska et al. [46] proposed major and minor dermoscopic criteria for the diagnosis of FPHL. Major criteria include (1) more than 4 yellow dots in 4 images in the frontal area; (2) lower average hair thickness in the frontal area compared with the occiput; and (3) more than 10% of thin hairs (<0.03 mm) in the frontal area. Minor criteria include (1) increased frontal to occipital ratio of single-hair pilosebaceous units; (2) vellus hairs; and (3) perifollicular discoloration. The diagnosis of FPHL is made during the presence of two major criteria or one major plus two minor criteria.

### 7.6. Pull Test

Pull test is a noninvasive diagnostic technique, very easy to perform and to repeat. Pull test is very helpful to rapidly determine the ongoing activity and severity of any kind of hair loss. Briefly, a bundle of about 50–60 hairs is grasped between the thumb, index finger, and middle finger from the base near the scalp. The hair is firmly, but not forcibly, tugged away from the scalp as fingers slide along the hair shaft [47]. The test is positive when more than 10% of the grasped hair (in average more than six hairs) can be pulled out [48]. If fewer than six hairs can be easily pulled out, this is considered normal physiologic shedding [49]. The test has a large interobserver variation and can be influenced by cosmetic habits, hair manipulation, and shampooing. Each clinician has to standardize his own procedure. The pull test should be done in all the scalp areas: right and left parietal, frontal, and occipital regions. A positive test present in more than one scalp region can be seen during a telogen effluvium. The patients suffering from FPHL may have a positive pull test only during the active phases in the affected area. A diffuse positive pull test requires always further investigation to exclude telogen effluvium [23].

## 8. Other Diagnostic Techniques

### 8.1. Scalp Biopsy

Scalp biopsy is an essential instrument in the diagnosis of cicatricial and selected forms of noncicatricial alopecia [50]. Although scalp biopsies are usually not needed to diagnose FPHL, they can be helpful if the clinical evaluation does not provide a definitive diagnosis, for example, when scalp changes suggestive of cicatricial alopecia of diffuse alopecia areata are present. A 4 mm punch extending into the subcutaneous fat should be performed on the central scalp area. It is best to avoid the bitemporal area as this region may have miniaturized hairs in women without hair loss [23]. Scalps biopsies should be read by experienced dermatopathologists using both vertical sectioning and horizontal sectioning. Horizontal sections allow a rapid evaluation of hair follicle number, diameter, grouping, and morphology maximizing the diagnostic yield [51]. In FPHL, there are an increased number of miniaturized (vellus-like) hairs. The ratio of terminal to vellus-like hair follicles is typically >3 : 1 in women suffering from this condition against >7 : 1 in the normal scalp [14]. Other typical histopathological features are an increase of telogen : anagen ratio and an increased number of follicular stelae. A mild perifollicular inflammation around the upper portion of hair follicle as well as perifollicular fibrosis may also be seen [52].

### 8.2. Trichogram

The trichogram is a semi-invasive (plucking) microscopic method for hair root and hair cycle evaluation. The term “trichogram” was given by Pecoraro et al. in 1964, who described further trichometric parameters such as hair shaft diameter, hair growth, and telogen rate [53]. The trichogram is based on the hair cycle and quantifies hair follicles in their different growth phases: anagen, telogen, or catagen. Trichogram may be recommended in individual cases of FPHL if another diagnosis is suspected like an anagen-dysplastic effluvium or a loose anagen syndrome. Trichogram can also be useful as a complementary element that may confirm an early diagnosis of FPHL by showing inhomogeneous hair shafts. However, a recent study suggests that dermatoscopy is more useful for the diagnosis of FPHL than trichograms [54].

### 8.3. Phototrichogram/TrichoScan

Phototrichograms are automatic digitalized imaging techniques used to examine features of hair loss for diagnosis and followup [55]. A small area of the scalp is trimmed and followed with imaging. The proportion of anagen, telogen, and shed hairs as well as the rate of growth and the density can be recorded and compared [56]. The FPHL is characterized, like AGA, by a decrease in the frontal hair density compared with the occipital density. To ensure reproducibility tattoos identifying the studied area are required [48]. When available, those techniques are helpful for long-term followup and quantification, but currently they are mainly used as a tool for clinical studies [57].

### 8.4. Laboratory Tests

Ferritin and thyroid-stimulating hormone levels may be measured, especially in diffuse effluvium. The association between FPHL and low ferritin levels was suggested in two different studies [58, 59] which reported significantly lower ferritin levels in women suffering from FPHL compared with controls. More recent studies have not shown sufficient evidence of the relationship between low ferritin level and FPHL and do not recommend iron supplementation in the absence of deficiency anemia [60]. In 2008, Bregy and Trüeb even suggest no association between iron deficiency and hair loss in women [61]. The experts agreed that an extensive endocrinological investigation is not necessary in all women. Research for hyperandrogenic state will be performed in women with FPHL when the history and the clinical examination are suggesting an androgen excess (e.g., hirsutism, irregular menses, acne, and galactorrhea). Some authors speculate that the Hamilton IV pattern is more common in women suffering from ovarian hyperandrogenism, but there are no studies to corroborate or invalid this theory. The authors recommend a free androgen index test (FAI = total testosterone (nmolL^−1^) × 100/sex hormone-binding globulin (SHBG)) and prolactin as screening parameters for ovarian hyperandrogenism and dehydroepiandrosterone sulfate (DHEAs) and 17-hydroxyprogesterone (17-OH) as screening parameters for surrenal hyperandrogenism [23, 39, 62]. Depending on the results, further investigations may be needed and an interdisciplinary approach involving gynecologists, endocrinologists, and dermatologists may be required. Note that androgen levels testing should be done during the follicular phase, between the fourth and the seventh day of the cycle and that oral contraceptives should be discontinued for at least two months prior to this testing [63]. The patients should be informed that cessation of contraception may induce a three-month lasting telogen effluvium.

## 9. Summary

FPHL is a very common, nonscarring form of hair loss that can occur in all ages but most commonly in postmenopausal women. Although hormonal factors and genetic predisposition are believed to contribute to FPHL, the complete mechanism remains elusive and the most affected women have normal androgen levels. FPHL does not cause physical discomfort but the hair loss can contribute to significant psychological distress. Generally, the condition is clinical diagnosis, suggested by the reduction in hair density with a characteristic distribution. Depending on patient history and clinical evaluation, further diagnostic tests may be required.
